# Intracellular Trafficking of Baculovirus Particles: A Quantitative Study of the HearNPV/HzAM1 Cell and AcMNPV/Sf9 Cell Systems

**DOI:** 10.3390/v7052288

**Published:** 2015-05-05

**Authors:** Leila Matindoost, Lars K. Nielsen, Steve Reid

**Affiliations:** Australian Institute for Bioengineering and Nanotechnology, University of Queensland, St Lucia, QLD 4072, Australia; E-Mails: lars.nielsen@uq.edu.au (L.N.); steven.reid@uq.edu.au (S.R.)

**Keywords:** baculovirus, HearNPV, AcMNPV, HzAM1, Sf9

## Abstract

To replace the *in vivo* production of baculovirus-based biopesticides with a more convenient *in vitro* produced product, the limitations imposed by *in vitro* production have to be solved. One of the main problems is the low titer of HearNPV budded virions (BV) *in vitro* as the use of low BV titer stocks can result in non-homogenous infections resulting in multiple virus replication cycles during scale up that leads to low Occlusion Body yields. Here we investigate the baculovirus traffic in subcellular fractions of host cells throughout infection with an emphasis on AcMNPV/Sf9 and HearNPV/HzAM1 systems distinguished as “good” and “bad” BV producers, respectively. qPCR quantification of viral DNA in the nucleus, cytoplasm and extracellular fractions demonstrated that although the HearNPV/HzAM1 system produces twice the amount of vDNA as the AcMNPV/Sf9 system, its percentage of BV to total progeny vDNA was lower. vDNA egress from the nucleus to the cytoplasm is sufficient in both systems, however, a higher percentage of vDNA in the HearNPV/HzAM1 system remain in the cytoplasm and do not bud out of the cells compared to the AcMNPV/Sf9 system. In both systems more than 75% of the vDNA produced in the nuclear fraction go unused, without budding or being encapsulated in OBs showing the capacity for improvements that could result from the engineering of the virus/cell line systems to achieve better productivities for both BV and OB yields.

## 1. Introduction

Baculoviruses are a family of circular dsDNA insect-specific viruses of which two genera, *Alphabaculovirus* and *Betabaculovirus*, infect larvae of Lepidoptera. *Alphabaculoviruses* which are known as nucleopolyhedroviruses have many applications including being used as biological control agents for lepidopteran pests [1], as vectors for protein expression [2], or virion display [3,4,5], and as gene delivery vectors for transducing mammalian cells [6].

To scale up baculoviruses *in vitro* for any of the above applications, commercially, there is a need for high budded virus (BV) titers. However for some baculoviruses, such as *Helicoverpa armigera* Nucleopolyhedrovirus (HearNPV), due to the low BV titers that they produce; their commercial production as a biopesticide is in jeopardy as the performance of baculovirus bioprocesses largely depends on an efficient infection of cells by concentrated BV inoculums. Budded virions start infections via attachment to the cell surface by the receptor binding activity of the viral envelope fusion proteins (EFP) [7]. EFP plays a major role in the budding, binding and internalization of the virions, hence, *Alphabaculoviruses* are distinguished on the basis of their EFP into two phylogenetic groups, I and II [8]. The EFP for group I is GP64 and for group II it is referred to as the F protein. GP64 and F protein have structural and functional differences and it has been hypothesized that *gp64* is a recent development by Type I viruses conferring a selective advantage for them in terms of binding and budding [9]. Therefore, higher BV titers of group I baculoviruses, such as *Autographa californica* Multiple Nucleopolyhedrovirus (AcMNPV), that produce virus titers of 10^8^ to 10^9^ PFU/mL [10,11,12], compared to HearNPV, a group II baculovirus demonstrating titers often as low as 1–2 × 10^7^ PFU/mL [13], has been attributed, at least in part to the higher efficiency of the GP64 protein in terms of binding and entering the cells and also aiding the subsequent budding process [13]. However reports of group II baculoviruses producing high titers as is observed for group I baculoviruses, such as SeMNPV and HzSNPV have been published [14,15]. Other studies have also shown that the host cell line has as much influence on BV titers as virus phylogenetics [16] and the role of *gp64* might have been exaggerated as the key component of BV production efficiency [17]. Furthermore, in the study of Cheng *et al.* (2013), reduction of *polyhedrin* mRNA and protein expression levels in Sf9 and Hi5 cell lines, but not in Sf21 cells, infected with AcMNPV *fp25k* mutants indicated that *polyhedrin* gene expression activities are also influenced by different host cell lines [18], suggesting that the cell line can influence significantly the virus phenotype.

BV production is a complex procedure that involves many viral and cellular factors and although there has been a wealth of data published regarding the viral genes involved in BV production [12,19,20,21,22,23] and also BV binding, endosmal sorting and internalization [7,24], when studying the processes after vDNA replication, the paucity of detailed knowledge of these events is realized. It is not clear how virions are distributed inside the cells quantitatively and how many of the vDNA exit the nucleus to the cytoplasm or what percentage leaves the cytoplasm and bud out of the cells. As was demonstrated earlier [17], HearNPV infected-HzAM1cells released a lower percentage of vDNA to the extracellular fraction when compared to Sf9 cells infected by AcMNPV. A quantification of vDNA populations in different fractions of the cell throughout the infection will provide some insight into the efficiency of the movement of virions out of the nucleus and out of the cell by Type II *versus* type I viruses. In addition, as the previous study indicated that the HzAM1/HearNPV system manages to bud out similar vDNA/cell as the Sf9/AcMNPV system, it is important to investigate why the infectious BV titer levels of HearNPV are 10–100 fold lower than that seen for AcMNPV [17]. In other words, the Hzea/HearNPV system appears to produce a lower Infectious BV/Total BV (BV_I_/BV_T_) ratio than the Sf9/AcMNPV system.

Genetic alterations of passaged virus, such as defective interfering particle (DIP) mutants, which are replication-defective deletion mutant viruses that arise during passaging and compete with the production of the normal wild-type virus [25,26] could cause reduced virulence of virions, and are expected to be one cause for the low ratio of infectious to total virions for some viruses. To investigate DIP accumulation in passaged HearNPV, pulsed-field gel electrophoresis (PFGE) of extracted extracellular virus DNA *in situ* and restriction enzyme digestion at a single site for linearizing the circular genome was used to compare the genome size at different passage numbers and also transmission electron microscopy (TEM) images were used to measure the length of virions at a number of passages [27].

The current study has conducted an investigation of viral DNA (vDNA) content in the different fractions of HearNPV (a group II baculovirus) and AcMNPV (a group I baculovirus) infected host cells to investigate possible differences in the pattern of vDNA distribution for these two viruses, which may reflect general differences between the two phylogenetic groups of baculoviruses that they represent. Western blot analysis of appropriate marker proteins was used to show a clean fractionation of infected cells without any leakage of the nuclear and cytoplasm contents. PFGE and TEM imaging were also used to monitor any defects in the viral genome and virions, respectively of the early passages of the HearNPV baculovirus, which might account for the high extracellular vDNA/PFU (Plaque Forming Units) ratio for this virus in culture.

## 2. Materials and Methods

### 2.1. Viruses, Cell Lines, and Medium

The Sf9 and HzAM1 cell lines cultured in SF900III SFM (Gibco®, Life technologies, Carlsbad, CA, USA) were incubated at 28 °C in 250 mL flasks shaking at 120 rpm on an orbital shaker. BV stocks of r-β-gal AcMNPV (a recombinant AcMNPV virus expressing the B-Galactosidase gene) [28] and HearNPV were prepared as described in Matindoost *et al.* (2012) [29]. Cell number and size distribution were determined using a Multisizer™ 4 Coulter Counter^®^ (Beckman Coulter, Brea, CA, USA).

### 2.2. Infection Set Up

Infections were prepared in 100 mL cultures seeded with 0.5 × 10^6^ and 1 × 10^6^ mid-exponential cells/mL of HzAM1 and Sf9 cells, respectively. Passage 3 viruses were used to conduct synchronous infections with an MOI of 10 for both AcMNPV and HearNPV experiments. Three biological replicates were used for each cell line/virus combination. Whole culture samples were taken at 6 hourly time points from 0 hpi for up to 42 hpi, while the cell viability remained higher than 90%. 48 h and 72 h samples were taken to determine the occlusion body vDNA content.

### 2.3. Cell Fractionation into Cytoplasmic and Nuclear Fractions

To fractionate the cells into cytoplasmic, nuclear and OB fractions, the cell pellet was re-suspended in 100 µL of ice-cold NP40-lysis buffer (10 mM TRIS-Cl at pH 8.0, 140 mM NaCl, 1.5 mM MgCl_2_, 0.5% Nonidet-P40) and incubated on ice for 5 min with periodic gentle inversion of the tube. The complete lysis of all cells by the Nonidet-P40 detergent, but not of the nuclei, was verified by monitoring the cells under a microscope. Nuclei were pelleted at 2000 g for 5 min and the cytoplasmic extract was digested with digestion buffer (0.5% SDS, 0.2 g L-1 Proteinase K, 100 mM NaCl, 25 mM EDTA, 10 mM TRIS-Cl, pH 8.1) at 28 °C for 30 min to release the vDNA. Pelleted nuclei were washed 3 times with medium to eliminate any vDNA or material from the cytoplasmic fraction. The nuclei were then digested by 500 µL of digestion buffer at 55 °C for 1h to release the remaining virus genomes. In the case of HearNPV, to enumerate the genomes in the OB fraction, the nuclei digest was centrifuged at 5000 *g* for 15 min to pellet the OBs. The OB pellet was washed with MilliQ water 3 times and then was re-suspended in 500 µL of water and 50 µL of alkaline saline (25 mM Na_2_CO_3_, 50 mMNaCl), which was added to extract the virion genomes. Fractions were diluted with injection water 10^−2^ to 10^−4^ and were subjected to Real Time-quantitative-PCR.

### 2.4. Western Blotting

For Western blotting, extracts of cytoplasmic and nuclear fractions of 1 × 10^6^ cells/mL infected by virus at an MOI of 10 PFU/cell were resolved by 12% SDS-PAGE. The protein bands were transferred to a PVDF membrane (Invitrogen, Carlsbad, CA, USA) and blocked with 20 mM Tris-HCl, pH 7.5, 500 mM·NaCl, 5% non-fat milk, 0.1% (*w*/*v*) Tween-20 for 1 h. The membranes were probed by a mouse monoclonal anti-GAPDH (Thermo scientific, Rockford, IL, USA) to detect glyceraldehyde-3-phosphate dehydrogenase (GAPDH), an enzyme that plays a role in glycolysis, as a specific cytosolic marker to exclude cytoplasmic contamination of the nuclei preparation. Histone H3, as one of the five main histone proteins involved in the structure of chromatin in eukaryotic cells, served as a specific nuclear marker to exclude nuclear leakage into cytoplamic samples and was visualized by using a rabbit monoclonal anti-Histone H3 antibody (Thermo scientific, Rockford, IL, USA). GAPDH and Histone H3 had previously been used as cytosolic and nuclear markers, respectively [30,31].

The membrane was first incubated with the GAPDH and the Histone H3 antibodies raised against the GAPDH and the Histone H3 proteins (1:5000) followed by incubation with horseradish peroxidase-coupled goat anti-mouse IgG and goat anti-rabbit lgG (1:2000) secondary antibodies (Thermo scientific, Rockford, IL, USA) respectively. An ECL solution (Thermo scientific, Rockford, IL, USA) was used to allow the horseradish peroxidase to catalyse its substrate, which released a detectable signal. The protein bands were visualized by a CCD camera system (VersaDoc MP 4000 system, Bio-Rad, CA, USA). Duplicate analyses were conducted for samples taken at 45 hpi.

### 2.5. Real-Time Quantitative PCR

To quantify viral genomic numbers of different cell fractions two fluorescent TaqMan^®^ Probes that target the DNA polymerase genes of the wild-type HearNPV (HanDNApol) and r-β-gal AcMNPV were used. TaqMan^®^ fluorogenic probes technology uses the 5'exonuclease activity of Taq polymerase to digest the probe, hybridized between flanking PCR primers, and labeled with two fluorescent dyes. Quantitative PCR was performed using previously developed probes and primers in a 7900 HT (Applied Biosystems, Foster City, CA, USA) instrument following the protocol that was described earlier [17]. Briefly, PCR reactions were performed as follows: initial denaturing at 95 °C for 10 min, followed by 50 cycles of denaturing at 95 °C for 15 s, annealing and extension at 60 °C for 1 min, final extension step at 60 °C for 10 min. Statistical analysis of qPCR data was carried out using triplicate cycle threshold (CT) values as determined by automated threshold analysis with ABI Prism version 1.3 software. The actual DNA content was evaluated from a standard curve prepared with purified DNA. This standard curve allowed the determination of virus concentrations from various samples by extrapolation from a standard curve of virus DNA content *vs.* CT. A clear distinction between the levels of virus in different samples could be made on the basis of their CT value. Genome copy numbers of HearNPV (131 kbp) and AcMNPV (133 kbp) were obtained by conversion factors of 1.4 × 10^−16^ and 1.359 × 10^−16^ g/genome, respectively, based on their genome size. DNA standards were prepared from BV supernatants and purified using a QIAamp Tissue Kit (QIAGEN, Valencia, CA, USA). The DNA standard concentrations were determined using Quant-iT™ PicoGreen (Invitrogen, Carlsbad, CA, USA).

### 2.6. Pulse Field Gel Electrophoresis (PFGE)

Pulse field gel electrophoresis was performed to monitor the formation of DIPs in early passages of the wild type HearNPV virus. The method of Giri *et al.* 2012 was followed [27]. Fifty milliliter cultures of various BV samples were pelleted by ultra-centrifugation at 100,000 *g* for 1 h at 4 °C. The resulting pellet of around 7.5 × 10^10^ vDNA was re-suspended in 1% low melting agarose in a final volume of 240 µL and poured onto block moulds. Large DNA is very easily sheared and often difficult to pipet due to its high viscosity. Thus, Chromosomal DNA must first be embedded in agarose plugs and these plugs are treated with enzymes to digest the proteins surrounding and interacting with the DNA genome, leaving behind the naked DNA. The plugs are then cut to size, treated with restriction enzymes, loaded into the wells of the gel and sealed into place with agarose. The DNA of the BVs in the moulds was first extracted using a lysis buffer (200 µg/mL proteinase k and 1 µL RNAse mL^−1^ buffer) and incubated at 50 °C overnight.

Before restriction enzyme cuts were made, plugs were soaked two times in TE (10 mMTris, 1 mM EDTA) buffer (1 mL per wash for 1 h). TE buffer was replaced by 0.5 mL of restriction enzyme buffer (buffer 4, New England Biolabs, Ipswich, MA, USA) diluted 10× for 1 h. To digest the DNA, the plugs were incubated in 60 µL AscI (this enzyme cuts *orf56*, *cathepsin*, in a non hr position of the HearNPV genome), reaction buffer at 37 °C overnight. The DNA plugs were then inserted into the wells of a 1% pulse field certified agarose (Bio-Rad) gel in 0.5× TBE (40 mM Tris-Cl, pH 8.3, 45 mM boric acid and 1 mM EDTA). Lambda Ladder PFG Markers (New England Biolabs, Ipswich, MA, USA) were used as MW standards. The DNA was then separated by PFGE in a CHEF-DR III system (Bio-Rad, Gladesville, NSW, Australia) at 6 V cm^−1^ with an initial switch time of 5 s and a final switch time of 20 s for 23 h at 14 °C in 0.5× TBE buffer.

### 2.7. Electron Microscopy

HearNPV infected culture supernatants from passage 1 to passage 6 were ultracentrifuged at 100,000 *g* for 1.5 h to obtain the virion pellets of each sample. The re-suspended pellet in fresh medium was applied to carbon coated copper grids for 3 min and stained by Uranyl acetate. More than 50 virion particles from each sample were observed under a JEM-1010 transmission electron microscope (JEOL, Peabody, MA, USA) at an accelerating voltage of 100 kV. The lengths were measured using the electron micrographs of the transmission electron microscope.

## 3. Results

### 3.1. Cell Fractionation

**Figure 1:**
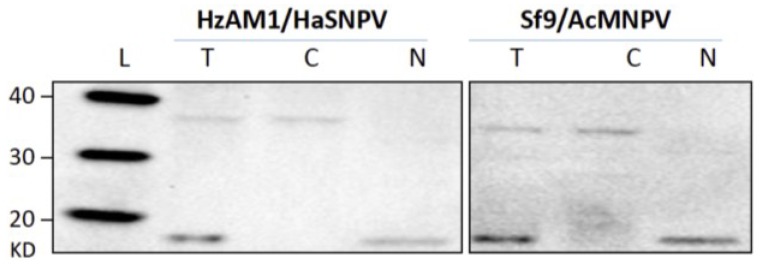
Western blot of nuclear and cytosololic fractions of the infected cell lines of HzAM1/HearNPV and Sf9/rAcMNPV at 45 hpi. GAPDH (37 kDa) was used as a cytoplasmic marker and Histone H_3_ (18 kDa) as a nuclear marker to assess contamination during cell fraction isolation. (L) Magic Mark western protein standard. (T) Total cell lysates of infected cells. (N) Nuclear fraction and (C) cytoplasmic fraction. For the HzAM1/HearNPV system, nuclei, cytoplasm and total cell lysates of 0.625 ×10^5^ cells were applied to each lane and for the Sf9/AcMNPV system, nuclei, cytoplasm and total cell lysates of 2.5 ×10^5^ cells were applied to each lane.

To quantify vDNA populations in the cytoplasmic and nuclear fractions, nuclear and cytosolic fractions were prepared from infected HzAM1 and Sf9 cells. Western blot assessments of these fractions showed dissociation of the cytoplasm content from the nuclear content without contamination between the fractions up to 45 hpi (Figure 1). GAPDH with a 37 kDa molecular weight was used as a specific cytosolic marker to exclude any cytoplasmic contamination of nuclear samples during preparation and reversely, the purity of the cytoplasmic fraction was validated by use of an antibody against the nuclear marker protein Histone H3 (18 kDa). The Western blotting analyses using the total cell lysate suggested that both markers were sensitive enough to detect any cross contamination.

### 3.2. Subcellular Populations of vDNA in Host Cells

To investigate the limitations of BV production in the HzAM1/HearNPV system compared to the Sf9/AcMNPV system, we studied the vDNA populations in different fractions of the two host cells. To this end, HzAM1 and Sf9 cells were infected with a high MOI of 10 to achieve a synchronous infection with very low cell growth post-infection. Cell fractionation was pursued using a cell membrane specific detergent (NP40 buffer) to separate the cytoplasm content from the nuclear content and a digestion buffer was used to further extract the nuclear content and finally an alkaline saline solution was used to extract the vDNA content present in OBs resulting from the HearNPV infection. The progeny vDNA levels (assumed to reflect the virion content) of each extract was then measured by q-RT-PCR as outlined in the methods section.

**Figure 2:**
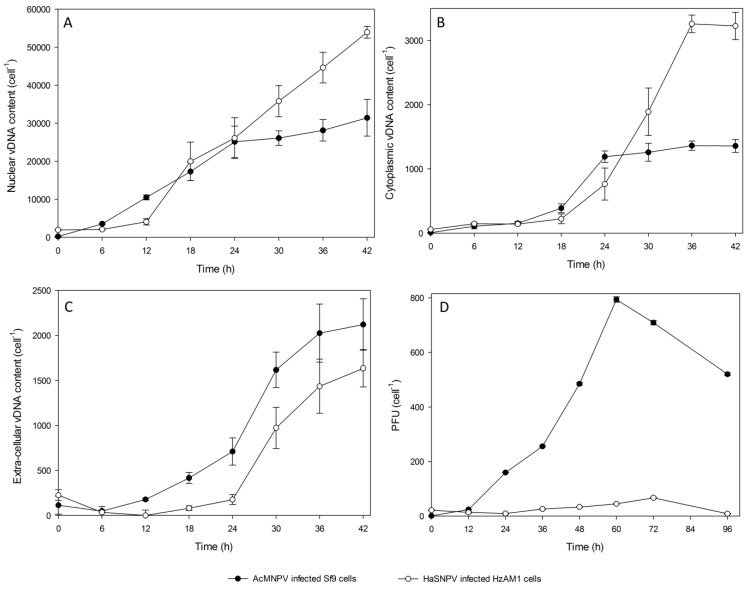
Cell specific production kinetics of HearNPV infected HzAM1 cells and AcMNPV infected Sf9 cells. (**A**) Nuclear vDNA levels; (**B**) Cytoplasm vDNA level; (**C**) Extracellular vDNA levels; (**D**) Budded virus production. The multiplicity of infection was 10 PFU per cell for all infections. HzAM1 cells were infected at 0.5 × 10^6^ cells per mL and reached a peak cell density of 0.55 × 10^6^ cells per mL. Sf9 cells were infected at 1.0 × 10^6^ cells per mL and reached a peak cell density of 1.06 × 10^6^ cells per mL. Each data point is an average of three biological replicate cultures. The error bars represent the standard deviation interval.

To more thoroughly characterize the vDNA distribution, the vDNA content in the nuclear, cytoplasmic and extracellular fractions of host cells were determined by q-RT-PCR at various times post infection (Figure 2A–C). The time course analysis showed that following HearNPV infection, vDNA replication increased slightly from 6 to 12 hpi and it then increased at a significant rate until 42 hpi. As cell lysis started to occur after 42 hpi, further analysis of the nuclear and cytoplasmic vDNA content was not conducted after this time. The onset of DNA replication occurred between 0 and 6 hpi for all biological replicates of the AcMNPV infections and continued to increase at a significant rate until 24 hpi followed by a further slight increase until 42 hpi. Progeny virion production of Sf9 cells (approximately 30,000 vDNA/cell) was 1.5–2 fold lower than that by HearNPV infected HzAM1 cells at 42 hpi (Figure 2A).

Interestingly, the qPCR data was sensitive enough to detect vDNA in the cytoplasm of HearNPV and AcMNPV infected cells within the first 6 h of infection. At 6 hpi, 87 ± 14 vDNA were detected in the cytoplasm of HzAM1 cells while only 21 ± 4 vDNA were measured in the AcMNPV infected cytoplasm at this time (Table 1 and Table 2). HearNPV progeny virions were unlikely to have been exported from the nucleus to the cytoplasm of HzAM1 cells until after 6 hpi, as the total vDNA at 6 hpi was not significantly different to that seen at 0 hpi (Table 1). By 18 hpi 223 ± 76 vDNA/cell were present in the HzAM1 cytoplasm and the number of vDNA in the cytoplasmic fraction kept rising for up to 36 hpi (peaking at 3260 ± 140 vDNA/cell) (Table 3). For AcMNPV, progeny vDNA production and trafficking shows a slightly faster kinetics compared to that of HearNPV. This is supported by the fact that the total vDNA present in the Sf9/AcMNPV system at 6 hpi has significantly increased compared to that present at 0 hpi (209 *versus* 56, respectively, as shown in Table 2). This is also supported by the fact that by 6 hpi only 4% of the total virions added at the time of infection are left outside the cell for the Sf9/AcMNPV system compared to 40% for the HzAM1/HearNPV system. Of course the percentage of vDNA left outside the cell may be influenced by the total number of vDNA added at the Time of Infection. For the HearNPV system 300–360 virions were added (due to the high ratio of 30 extracellular vDNA/BV_INF_) per cell *versus* only ~56 vDNA/cell for the AcMNPV system (ratio of 5 extracellular vDNA/BV_INF_). Due to cell lysis from around 40–45 hpi, the cytoplasmic vDNA content was not measured after 42 hpi.

In general, Figure 2A indicates that after 6 hpi vDNA replication by HearNPV in the cell nucleus exceeded that by AcMNPV to a slight degree until 24 hpi, after which HearNPV vDNA continued to increase while AcMNPV vDNA numbers levelled off in the nucleus. Figure 2B indicates that cytoplasmic vDNA increased at a slightly faster rate for AcMNPV than for HearNPV until 24 hpi after which HearNPV vDNA numbers continued to increase in the cytoplasm, while again vDNA numbers in the cytoplasm levelled off for AcMNPV. This data reflects a more efficient process of exporting vDNA from the cytoplasm to outside the cell by AcMNPV compared to HearNPV. After 24 hpi, it is possible that the rate of vDNA release from the nucleus to the cytoplasm is matched by the rate of vDNA release from the cytoplasm to outside the cell for AcMNPV, while for HearNPV, vDNA accumulate in the cytoplasm until the cells start to lyse due to a less efficient cell budding process.

**Table 1:** Distribution of HearNPV vDNA in various cell fractions during the first 30 hpi of HzAM1 cells at MOI of 10.

Time (hpi)	Nuclear vDNA/cell	Cytoplasmic vDNA/Cell	Extracellular vDNA/cell	OB vDNA/cell	Total progeny vDNA	Nuclear % of total	Cytoplasmic % of total	Extracellular % of total	OB % of total
0	2 ± 2	60 ± 13	279 ± 52	0	341	1	17	82	0
6	134 ± 14	87 ± 14	137 ± 53	2 ± 1	360	37	24	38	1
12	6667 ± 432	94 ± 10	129 ± 72	62 ± 18	6952	96	1	2	1
18	19,967 ± 5069	223 ± 76	282 ± 127	738 ± 125	21,210	94	2	1	3
24	26,279 ± 6082	764 ± 250	400 ± 28	1247 ± 320	28,690	92	3	1	4
30	35,837 ± 4594	1891 ± 369	1128 ± 218	3485 ± 866	42,341	85	4	3	8

**Table 2:** Distribution of AcMNPV vDNA in various cell fractions during the first 30 hpi.

Time (hpi)	Nuclear vDNA/cell	Cytoplasmic vDNA/cell	Extracellular vDNA/cell	Total progeny vDNA	Nuclear % of total	Cytoplasmic % of total	Extracellular % of total
0	10 ± 6	17 ± 0	29 ± 10	56	18	30	52
6	186 ± 100	21 ± 4	2 ± 1	209	89	10	1
12	4606 ± 634	153 ± 30	168 ± 14	4927	94	3	3
18	17,306 ± 2403	389 ± 67	417 ± 61	18,112	96	2	2
24	25,146 ± 4113	1191 ± 78	711 ± 151	27,048	93	4	3
30	26,109 ± 1906	1259 ± 140	1618 ± 197	28,986	90	4	6

**Table 3:** vDNA/cell for various fractions of HzAM1 and Sf9 infected cells at 42 hpi *.

Cell line/virus	Fraction	Peak vDNA/cell
HzAM1/HearNPV	Nuclear	54,000 ± 1,500
HzAM1/HearNPV	Cytoplasm	3260 ± 140
HzAM1/HearNPV	Extracellular	1635 ± 206
HzAM1/HearNPV	OB	11,000 ± 558
**HzAM1/HearNPV**	**Total**	**69,895**
Sf9/AcMNPV	Nuclear	31,450 ± 4,835
Sf9/AcMNPV	Cytoplasm	1360 ± 70
Sf9/AcMNPV	Extracellular	2120 ± 285
**Sf9/AcMNPV**	**Total**	**34,930**

* Note: 42 hpi was the latest time point at which valid subcellular fractions could be prepared.

Of interest from the data in Table 1 and Table 2 is the suggestion that for both viruses some vDNA can be detected in the nucleus at 0 hpi. In the experiments conducted, virus is added at 0 hpi but it takes a few minutes to pellet cells and to collect supernatants, so it is possible this is enough time for some virions to bind, enter the cell and be delivered to the nucleus [32], although contamination of the nuclear fraction by vDNA from the cytoplasm cannot be excluded. It should be noted however that if the 0 hpi nuclear vDNA levels were simply due to contamination from the cytoplasmic vDNA the nuclear levels for HearNPV would be expected to be higher than for the AcMNPV samples as the cytoplasmic levels at 0 hpi were three times higher for HearNPV at this time (60 for HearNPV *versus* 17 for AcMNPV). The process of virus binding and uptake appears to be more efficient for AcMNPV than for HearNPV but by 6 hpi significant vDNA numbers are in the nucleus for the HearNPV system as well. Virion replication is clearly underway by 6 hpi for AcMNPV (total vDNA at this time is four times the vDNA levels added at 0 hpi, 209 at 6 hpi *versus* 56 at 0 hpi, Table 2). Indeed, within minutes post infection up to 50% of the virions may have entered the cell and up to 20% may have reached the nucleus (assuming the 0 hpi data for AcMNPV is reasonably accurate and the cytoplasmic and nuclear numbers are not simply contamination from the supernatant and cytoplasmic fractions respectively). This data is consistent with recent papers suggesting that 0 hpi samples from AcMNPV infected *T. ni* cells show signs of virus gene expression and host cell gene responses to the virus infection [33,34]. However little vDNA replication has occurred by 6 hpi for HearNPV as the total vDNA numbers for HearNPV at 6 hpi are not significantly higher than that at 0 hpi as indicated above (360 *versus* 341 virions/cell respectively, Table 1). However by 12 hpi vDNA replication by HearNPV has overtaken that by AcMNPV (Table 1 and Table 2).

Figure 2C (extracellular vDNA levels), demonstrates the quantities of vDNA in the culture supernatants. Initially this data shows the depletion of extracellular HearNPV, which was observed for the first 12 hpi, indicating the time needed for most of the virions added at the time of infection to be bound to receptors, taken up into the cells, and to enter the cytosol and nucleus, although these events only took 6 h, at most, in Sf9 cells infected by AcMNPV. For all infections, an MOI of 10 PFU/cell was used. However HearNPV has a vDNA/PFU ratio for extracellular virus of 27–30 [17], thus, the zero time total vDNA/cell reading of 341 is close to what would be expected. In general Figure 2C indicates that AcMNPV shows an increase of vDNA from 6 hpi while HearNPV only appears to release fresh vDNA from the cell after 12 hpi. vDNA numbers then appear to increase in a similar pattern for both viruses.

Table 3 indicates the peak vDNA/cell levels for various fractions of infected HzAM1 and Sf9 cells at 42 hpi. From this data, for the HzAM1/HearNPV system, around 4900 vDNA/cell leave the nucleus (sum of cytoplasmic and extracellular vDNA numbers), and of these approximately 66% remain in the cytoplasm and 33% bud out of the cell. However for the Sf9/AcMNPV system around 3500 vDNA leave the nucleus with about 39% remaining in the cytoplasm and 61% budding out of the cell. This data suggests HearNPV displays less efficient budding than AcMNPV.

The BV supernatants of HearNPV infected cells were assayed by a suspension culture titration method and an endpoint assay was used to determine infectious BV levels for AcMNPV infected Sf9 cells (Figure 2D). The BV production in HzAM1 cells showed a 12 h delay compared to the AcMNPV system with a gradual increase in titer culminating in a peak of 4 × 10^7^ PFU/mL at 72 hpi, (67 ± 1.3 PFU/cell). Titers of AcMNPV show a consistent increase from 12 hpi to 60 hpi and were much higher than that of HearNPV infected HzAM1 cells (up to 12 fold higher at 60 hpi, at around 800 PFU/cell). The vDNA/PFU ratio for each virus can be calculated from the peak extracellular vDNA levels from Table 3 divided by the peak PFU levels from Figure 2D. This data shows a vDNA/PFU ratio of 24.4 for HearNPV and 2.7 for AcMNPV suggesting the quality of AcMNPV BV is up to nine-fold better than for HearNPV (24.4/2.7).

**Figure 3:**
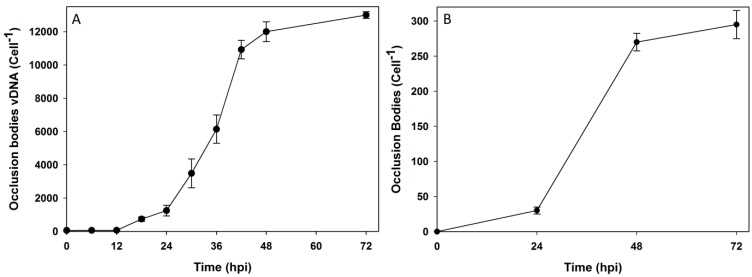
Cell specific genomic copies of the Occlusion body fraction within HearNPV infected HzAM1 cells (**A**) and Number of Occlusion bodies per cell within HearNPV infected HzAM1 cells at various time points (**B**). Each data point is an average of three biological replicate cultures. The error bars represent the standard deviation interval.

Occlusion body virion content was only measured for wild type HearNPV infected cells since the *polyhedrin* gene of the recombinant AcMNPV used in this study was knocked out and the virus is incapable of producing OBs. Progeny virions were packaged in OBs in significant numbers after 18–24 hpi and the OB vDNA content increased rapidly thereafter up to 48 hpi, with the peak content of occluded virions reaching 13,800 ± 200 vDNA/cell (Figure 3A). Figure 3B indicates that a peak of 306 OB/cell were produced, suggesting an average of around 45 virions were occluded per OB.

**Figure 4:**
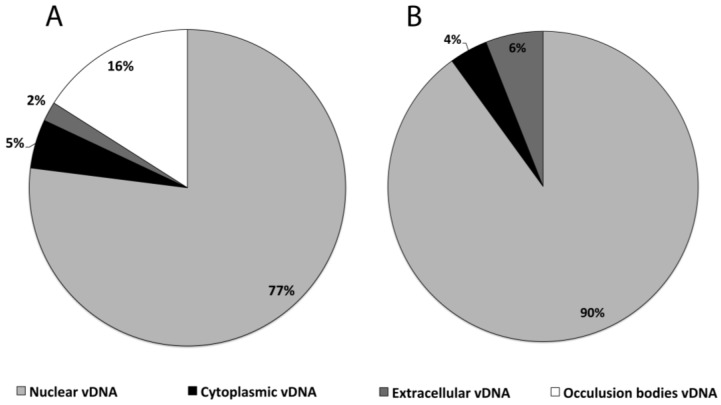
The population of vDNA in different sub-cellular fractions of insect cell lines as a percentage of total number of vDNA replicated by individual cells at 42 hpi. HzAM1 cells infected by HearNPV at an MOI of 10 PFU/cell at a cell density of 5 × 10^5^ cells/mL (**A**). Sf9 cells infected by a rAcMNPV at an MOI of 10 PFU/cell at a cell density of 1 × 10^6^ cells/mL (**B**).

As shown in the pie graph of Figure 4, the percentage of vDNA in the nuclear fraction of both cell lines is more than 75% of the overall vDNA produced showing that most of the vDNA are neither exported outside the cell nor packaged into OBs. These results also demonstrate that the vDNA content of HearNPV infected HzAM1 cells in the cytoplasm at 42 hpi corresponds to only 5.0% of the progeny vDNA produced and this number is about 4% for AcMNPV infected cells. AcMNPV show a higher percentage of extracellular vDNA (6.0%) compared to HearNPV (2%).

### 3.3. Transmission Electron Microscopy of HearNPV Virions

**Figure 5:**
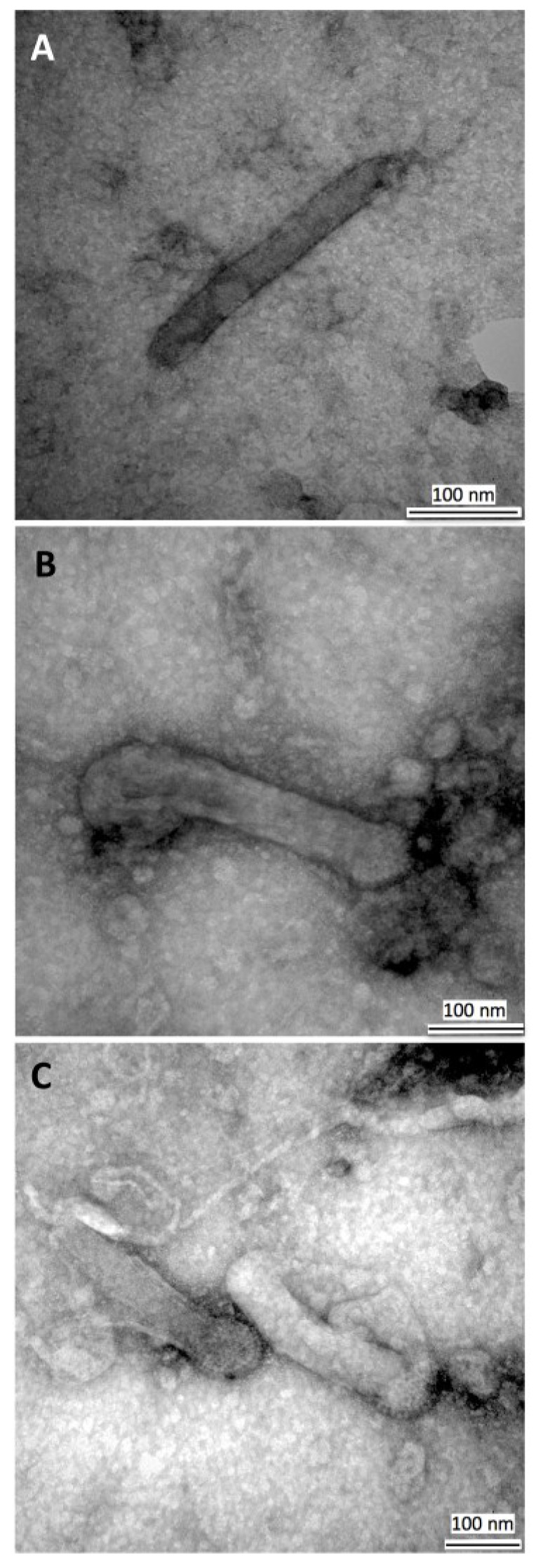
Transmission Electron Microscopy of HearNPV virions. Electron micrographs of wild type HearNPV BV particles at initial and late passages. P1, Passage 1, with standard size particles (**A**,**B**) and P6, passage 6 standard size particles (**C**). The size range of BVs remained constant through the first 6 passages of the virus.

**Figure 6:**
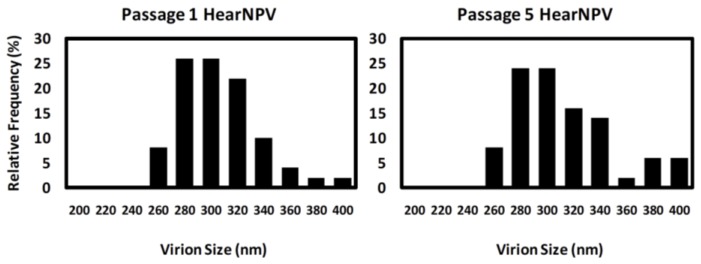
Size distribution of HearNPV BV particles at passages 1 and 5 obtained by TEM analysis (size interval, 20 nm). Relative frequency is the percentage of BV particles within a given size interval. The average BV length at passage 1 was 306 ± 30 nm (mean ± SD) and at passage 5 the average BV length was 312 ± 24 nm. A total of 50 BVs were measured per sample.

To investigate if there were any structural abnormalities in the BVs produced by HearNPV infections, extracellular virions were purified from infected cell media and observed under a transmission electron microscope (TEM) (Figure 5). Analyses showed the nucleocapsids to be rod shaped as expected and intact with distinct spike heads for up to six passages in culture. The size (length) distribution of standard particles in the HearNPV BV populations from passages 1 and 5 were examined and as shown in Figure 6 most of the virions show the expected size range of 280–320 nm (total of 75% frequency) which was consistent with that obtained by Giri *et al.*, 2012, for AcMNPV (size range of 288 ± 26) [27]. Although a slight size increase is observed for HearNPV virions at higher passages the data is not significantly different to that seen at the lower passages (Figure 6). These results indicate that HearNPV produces BV that appear to have a normal structure for up to six passages in culture, which is the passage range that would be used in a large scale production process to produce biopesticides *in vitro*. There was no sign from this TEM data suggesting the HearNPV were grossly abnormal in terms of size or structure. Hence the high vDNA/PFU ratios seen for the passage 3 HearNPV BV data shown in Figure 2, Figure 3 and Figure 4 and Table 1, Table 2 and Table 3 above, can not be explained on the basis of the formation of DIPs or other abnormal particles.

### 3.4. PFGE of HearNPV at Different Passages

**Figure 7:**
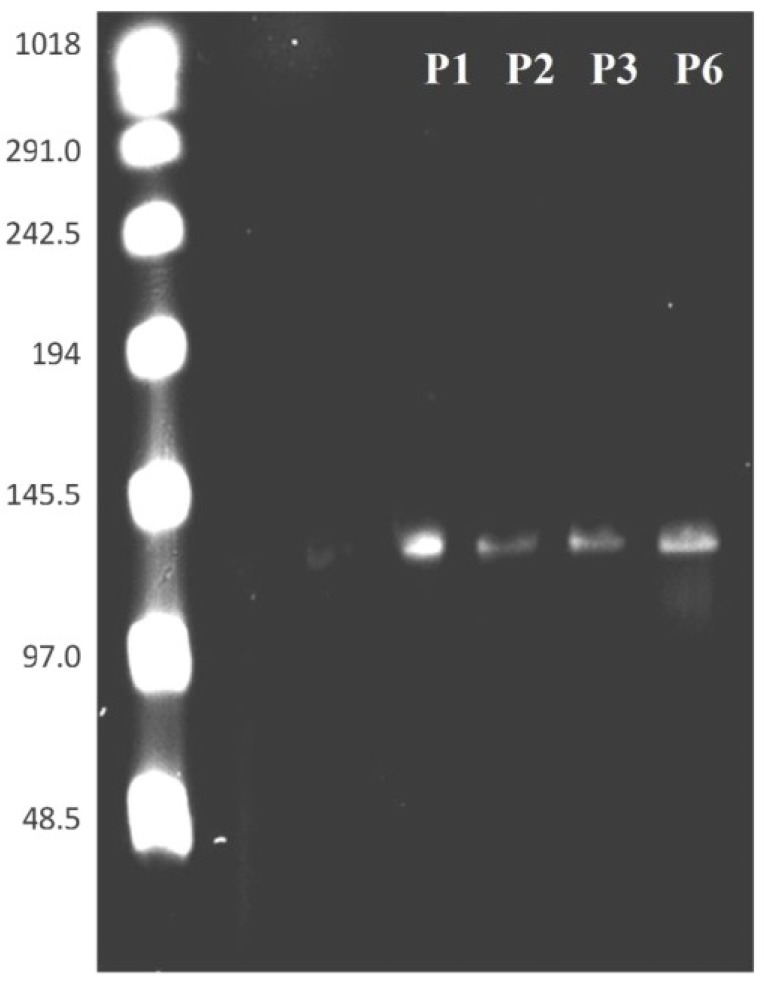
Detection and size distribution of HearNPV BV DNA linearized with *AscI* at different passages by Pulse Field Gel Electrophoresisi. DNA was obtained from purified budded virions. A Comparison of the virus genome from HearNPV DNA at passages 1 to 6 is shown. Lanes: L, Pulse Marker ladder (Sigma); L, DNA ladder, with sizes shown in kb (used as a control for electrophoresis and as a size marker); WT HearNPV DNA, P1, passage 1, P2, passage 2, P3, passage 3 and P6, passage 6.

Additional verification of the intactness of HearNPV DNA produced by HzAM1 cells was achieved by a single digestion of HearNPV DNA with the endonuclease *AscI*. This enzyme cuts WT HearNPV in the non hr location of *orf56* (*Cathepsin*) of the genome producing a single linear DNA form of the entire genome. As demonstrated in Figure 7, during the first six passages of HearNPV in culture, there are no extensive genome alterations and only single bands were observed following this treatment. A Sigma-Pulse Marker™ 50–1000 kb ladder (Sigma-Aldrich®, St. Louis, MO, USA) was used to determine the size of the genome and the expected size of 131 kb for the genome was confirmed [35]. Again this PFGE data indicates that the high vDNA/PFU ratio observed in this study for HearNPV is not due to DIP formation or major genome alterations occurring in the first six passages of the virus in culture as it was not consistent with PFGE analysis representing DIPs genomic DNA profiles of HearNPV as shown by [36].

## 4. Discussion

In this study the quantitative impact of vDNA levels produced and released into various host cell fractions on the levels of infectious BVs of a Type I and a Type II baculovirus were investigated. qPCR was used to compare the subcellular levels of viral DNA in (i) the prototypic and most intensively studied baculovirus with a broad host range of about 32 species, AcMNPV, (a type I baculovirus); and (ii) HearNPV, one of the major baculovirus pesticides to be introduced into the commercial marketplace which has a relatively narrow host range, (a Type II baculovirus).

Previous work by our group demonstrated that the low levels of infectious BV produced by HearNPV infected HzAM1 cells (10^7^ PFU/mL) compared to that produced by recombinant AcMNPV infections of Sf9 cells (10^8^–10^9^ PFU/mL) were not due to low levels of vDNA replication by HearNPV infections *versus* rAcMNPV infections [17]. Indeed HearNPV infected HzAM1 cells produce 2–4 fold higher vDNA levels than are produced by rAcMNPV infected Sf9 cells. However a number of possible explanations for the low infectious BV levels of HearNPV infected cells remain, ranging from poor transport of HearNPV DNA out of HzAM1 cells (either from the nucleus or from the cell or from both), to the generation of high levels of defective HearNPV DNA or simply the formation of HearNPV BV that are less stable in a culture supernatant compared to rAcMNPV BV. It is also possible that wild type baculoviruses “capture” so much vDNA for later occlusion, that less are available to bud out of the cell to form BV. As no quantitative data exists in this regard, it was possible that AcMNPV was more efficient at releasing vDNAs from the nucleus and applied a quality control to select only the most stable virions for export from the cell resulting in a low virion/PFU ratio. Hence, we investigated the process of vDNA release from infected cells in terms of efficiency of release from the nucleus to the cytoplasm and consequently from the cytoplasm to outside the cell for both the HearNPV/HzAM1 and AcMNPV/Sf9 systems.

The initial decrease in virus concentration in the medium is equal to the rate of virus attachment to the cells and changes in the extracellular virus concentration after initial progeny virion production (from 12 hpi on) are the outcome of the balance between budding and virus attachment rates. According to our data, AcMNPV is more efficient in binding and achieving entry to the Sf9 cells than the HearNPV/HzAM1 system, as 96% of AcMNPV virions added to Sf9 cells at the time of infection were attached to or taken up by the cells by 6 hpi while only 60% of the HearNPV virions present in the inoculum were attached to or taken up by the HzAM1 cells by 6 hpi (Table 1 and Table 2). These findings are consistent with previous studies that show attachment of more than 90% of AcMNPV virions added to Sf9 cells in the first few hours pi and a lower attachment (~50%) of the same virus to a cell line with a bigger size, such as Hi5 cells [7]. Because higher numbers of virions were added to HzAM1 cells compared to Sf9 cells, the presence of more HearNPV vDNA in the supernatant at 6 hpi of HzAM1 cultures may be attributed to an excess of vDNA over cell binding sites but, according to the model developed by Dee *et al.*, (1997), there is capacity for around 500 virus particles on the Sf9 cell surface at any one time while in the current study only ~341 HearNPV virus particles were added per HzAM1 cell (Table 1); therefore, in theory, the cell surface should not have been saturated by virus attachment (assuming HzAM1 cells have a similar number of virus binding sites as reported for Sf9 cells). Furthermore 11,000 receptors/cell have been estimated to be present on Sf21 cells for binding a baculovirus [7] but to our knowledge there are no reports of the number of baculovirus receptors on HzAM1 cells. However, it seems likely that AcMNPV and HearNPV will exploit different cell receptors given the fact they possess different envelope fusion proteins (*i.e.*, F protein for HearNPV or GP64 for AcMNPV) [37]. No doubt the number and distribution of virus receptors on a host cell line determines to a large extent the efficiency of infection.

As indicated above in reference to the data in Table 3, for HearNPV about 66% of the vDNA that leave the nucleus remain in the cytoplasm, while only 40% of the AcMNPV DNA that leave the nucleus remain in the cytoplasm. This is either due to slow dynamics of the vDNA egress rate for HearNPV or the inability of significant amounts of this virus to leave the cytoplasm because of a limitation of the EFP protein or deficiency in the EFP distribution throughout the cell membrane. While on a per cell basis more HearNPV leave the nucleus (around 4900 vDNA/cell), compared to AcMNPV (around 3500 vDNA/cell), if such data is considered on a unit mass basis, the efficiency of vDNA release for HearNPV is lower than for AcMNPV [17].

However, of more importance than a deficiency in egression, HearNPV virions suffer from low quality (high vDNA/PFU ratio). PFGE and electron microscopy (TEM) images (Figure 5, Figure 6 and Figure 7), confirm the intactness of the HearNPV extracellular genomes and BVs which make unlikely any conclusion about defective interfering particles being the cause of the high vDNA/PFU ratios for the HearNPV/HzAM1 system. The key difference between the nucleocapsid of budded virions of AcMNPV and HearNPV is their fusion protein. Previous studies have shown that enhanced expression of the HearNPV fusion protein via transient transfection or replacing the fusion gene with the AcMNPV *gp64* gene has not lead to higher BV titers in the HearNPV/HzAM1 system [38,39]. It is possible that the genes responsible for infectivity and transit of virions, including the host cell transport system, such as the endosomal sorting complex required for transport and F-actin and myosin motors are less efficient for the HearNPV/HzAM1 system than for the AcMNPV/Sf9 system [40,41]. 

Based on the TEM data of the current study and previous literature, the nucleocapsids of HearNPV and AcMNPV, have a length size range of 280–400 nm [7,27,39,42]. This large structure of the nucleocapsids restricts their free diffusion in the cytoplasm and their transit is mediated by a more directed process [43]. Despite the normal structure of most HearNPV BVs as shown by TEM (Figure 5 and Figure 6), the low BV titers observed for this virus could be due to lower expression of genes responsible for production of an efficient BV. Previous studies have shown that the absence of genes, such as *ac109* in AcMNPV allows the formation of BVs without any abnormalities in structure but lacking the ability to infect well [44]. The homologue of this gene in HearNPV is *Ha94*, and although the protein product from this gene is localized in HearNPV ODV, it has not been detected in HearNPV BVs either due to its low levels or its absence [44], which in either case means that AcMNPV BVs may benefit from proteins in their structure that enhance their infectivity that are at low levels or absent from HearNPV BV. The lower level of such proteins in HearNPV may lead to the high vDNA/PFU ratio seen for this virus. This could be due to impaired nucleocapsid assembly or inefficient egress of HearNPV BV [44].

It should be noted at this point that, in nature, the stability or quality of BV is possibly not as critical as it is for cell culture based production. In nature, BV leave infected cells into a high protein/slow flowing haemolymph environment and spend a short time in this environment before binding and entering another cell. However, in culture, the BV are thrust into a low protein highly mixed environment and are expected to survive relatively long periods before entering fresh cells. Cell densities in culture are very low compared to that present within a living organism. The challenge is to identify the reasons for this extra stability/quality for the AcMNPV BV in cell culture and to replicate it artificially for the HearNPV BV. This study supports the conclusion that the low PFU levels produced by HearNPV *in vitro* are not the result of inadequate vDNA replication and release or the formation of grossly malformed genomes and more likely result from the formation of unstable BV compared to AcMNPV BV.

In the current study, 77% of the HearNPV vDNA produced in the nuclear fraction appears to be unused, without budding or being encapsulated in OBs (Figure 4). This may demonstrate the capacity for improvements that could result from the engineering of the virus/cell line systems to achieve better productivities *in vitro*, for both BV and OB yields. However, it is possible the total vDNA measured in this study in the OB, cytoplasm, and extracellular fractions represent the total vDNA that is packaged in capsids (23% of the total vDNA measured), while the remaining 77% is non packaged vDNA that remains naked in the nucleus, available for transcription, in order to produce high levels of the virus late genes. Hence, the capacity to improve BV and OB levels for HearNPV *in vitro* may be limited by this need for very high levels of non-packaged vDNA.
